# Applying Molecular Dynamics Simulations to Unveil the Anisotropic Growth Mechanism of Gold Nanorods: Advances and Perspectives

**DOI:** 10.1021/acs.jcim.4c02009

**Published:** 2025-02-28

**Authors:** José Adriano da Silva, Paulo Augusto Netz, Mario Roberto Meneghetti

**Affiliations:** † Grupo de Catálise e Reatividade Química - GCaR, Instituto de Química e Biotecnologia, Universidade Federal de Alagoas, Av. Lourival de Melo Mota, s/n, CEP, Maceió, Alagoas 57072-900, Brazil; ‡ Instituto de Química, Universidade Federal do Rio Grande do Sul, Av. Bento Gonçalves, 9500, CEP, Porto Alegre, Rio Grande do Sul 91501-970, Brazil

**Keywords:** molecular dynamics, simulation, computational chemistry, surfactants, gold nanorods, surface chemistry, anisotropic growth mechanism, seed-mediated synthesis, metal nanoparticles

## Abstract

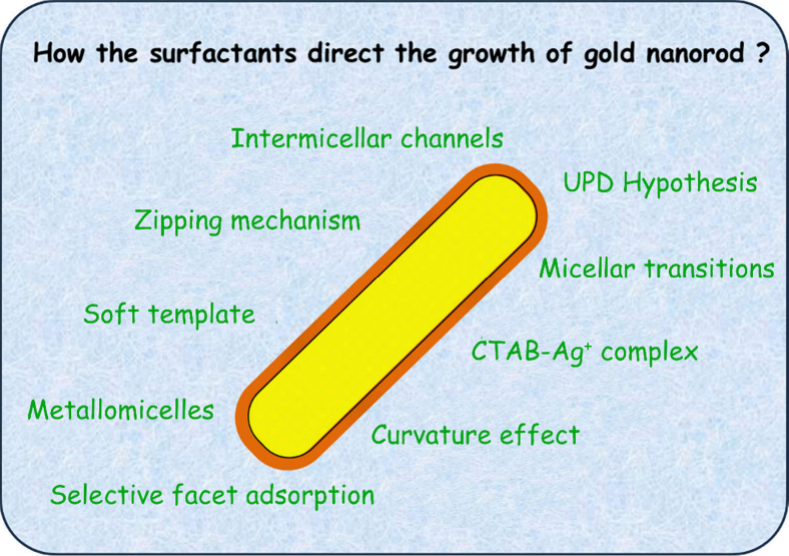

The unique properties of gold nanorods (AuNRs), combined with their relatively straightforward production, good yields, and satisfactory control over size and shape, have sparked considerable interest in their potential applications. However, the mechanism behind these particles’ formation continues to be a subject of significant interest and debate. Many experimental studies have been designed and undertaken to understand how AuNRs can be produced through seed-mediated methods. In recent years, quantum mechanics and molecular dynamics simulations have added to the repertoire of tools for investigating this topic. By comparing simulations with experimental data, essential aspects of the anisotropic growth of AuNRs can be revealed. This review presents an overview of the mechanisms proposed for creating AuNRs through seed-mediated methods, grounded in both experimental and simulation studies, and also highlights some remaining gaps in our understanding of the anisotropic growth process that need further exploration.

## Introduction

Though the preparation and utilization of gold nanoparticles (AuNPs) have been prevalent for centuries, significant advancements in fabricating anisotropic AuNPs with excellent yield, precise size, and shape control have only been achieved over the past three decades.^1,2^ Of these advancements, creating gold nanorods (AuNRs) using seed-mediated methods in aqueous media has attracted significant attention.^3^ This method was first established in 2001 by Murphy et al.,^4^ then later modified by El-Sayed’s team^5^ in 2003. Since its inception, it has been recognized as the traditional method for preparing AuNRs, offering the ability to produce particles with variable aspect ratios (ARs, length/width ratio) via minor adjustments to reaction conditions.

In the seed-mediated synthesis of AuNRs, the growth of these anisotropic nanoparticles occurs via the sequential addition of gold atoms to the surface of previously prepared small AuNPs (seeds). This process involves the controlled reduction of Au(I) species on the seed surface, driven by the presence of cetyltrimethylammonium bromide (CTAB) species adsorbed on the growing particle. Specific details of two standard seed-mediated synthesis methods for AuNRs are summarized in Table 1. Importantly, other studies and modifications of these standard synthesis methods^6^ have been proposed to explore the effects of varying parameters such as seed sizes,^7^ cosurfactant presence,^8,9^ additives,^10^ temperature,^11^ surfactant chain length,^12^ headgroup structure,^13−15^ the presence of polymers,^16^ alternative reducing agents to ascorbic acid, etc.^17,18^

**Table 1:** General Parameters for the Preparation of AuNRs Using Seed-Mediated Synthesis

Method of synthesis	Seed solution	Seed shape	Growth solution	Crystallinity of the AuNRs	AR^a^	Selectivity^b^
Murphy and co-workers^4^	HAuCl_4_ and citrate	Decahedral	Ascorbic acid, HAuCl_4_, and CTAB	Penta-twinned with pentagonal cross-section	6 to 20	ca. 15%
El-Sayed and co-workers^5^	HAuCl_4_, NaBH_4_, and CTAB	Cuboctahedral	Ascorbic acid, HAuCl_4_, CTAB, and AgNO_3_	Single-crystal with octagonal cross-section	1.5 to 5.0	ca. 90%

a AR: aspect ratio.

b Relative to the number of nanoparticles formed with a rod-like shape.

Despite the extensive literature on the production and application of AuNRs, some aspects and details of these nanoparticles’ formation in colloidal synthesis remain not fully understood. This is particularly true regarding the role of the surfactant, silver ions, and halides in the anisotropic growth process.

## The Anisotropic Growth Process of Rod-like Gold Nanoparticles in Colloidal Synthesis

**Figure 1:**
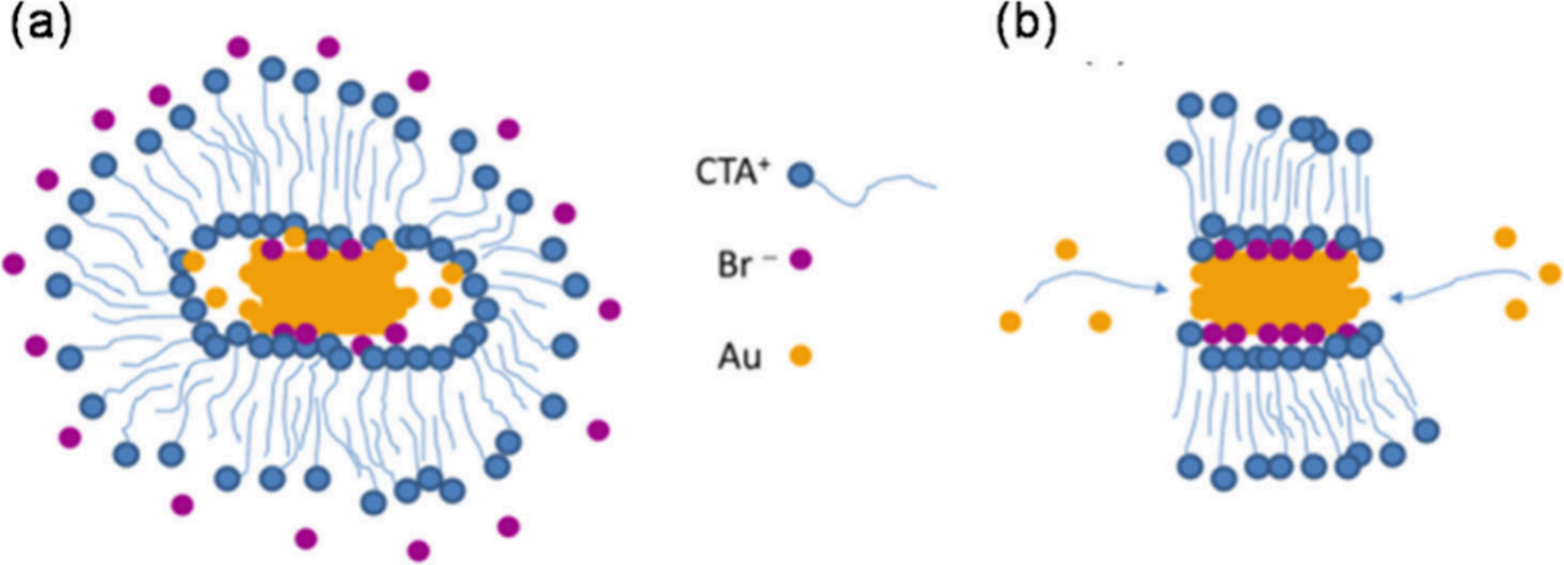
Illustration of (a) *soft template* and (b) *zipping* growth mechanism. Copyright 2018 American Chemical Society.^25^

Since the initial synthesis of AuNRs using seed-mediated methods, our comprehension of CTAB’s function in the anisotropic growth process of seeds into rod-like particles has progressed significantly. Early theories regarding this anisotropic growth suggested that the rod-like shape of CTAB micelles in the reaction medium could serve as a *soft template*, leading to the formation of AuNRs (either single-crystalline or pentatwinned) (Figure 1a).^19^ However, this hypothesis was soon dismissed since the seed particles, having diameters around 4–6 nm, are notably larger than CTAB micelles under the synthesis conditions. This makes it unlikely for micelles to serve as a *soft template* that envelops the particle and facilitates their growth.^20^ Furthermore, it was shown that AuNRs can be produced even at CTAB concentrations below the critical micellar concentration (CMC), provided the concentration of bromide ions remains at 0.1 M.^21^

In 2003, Murphy and colleagues proposed a mechanism alleging that CTAB species favorably adsorb onto the lateral surfaces of developing gold nanoparticles.^12^ They posited that when nanoparticles start to undergo anisotropic growth, a dense bilayer of adsorbed CTAB forms on the side facets of the growing particle. This formation restricts the access of the Au(I) species, the gold source, to these surfaces. Therefore, growth is favored on facets at the AuNRs’ tips, either single-crystal or pentatwinned, where the arrangement of the CTAB structure is sparser, allowing easier access for the gold source to the metal surface.^22^ This led to the conception of the so-called “*zipping* mechanism” (Figure 1b), which has since become a significant reference in literature for understanding the formation of AuNRs via seed-mediated methods. The mechanism was initially proposed based on thermogravimetric and infrared spectroscopic analyses of pentatwinned AuNRs, showing that CTAB assembles as a bilayer on the AuNR surface.^23^ Of noteworthy importance is the fact that when this mechanism was first suggested, it was understood that the primary driving force underlying the higher affinity of the polar CTAB head for the lateral facets of growing AuNR was linked to their relatively greater surface potentials. To put it simply, the facets designated as {100}, found on the lateral facets, possess a relatively larger potential surface owing to the lower coordination number of gold atoms exposed on that surface. Contrarily, on the {111} facets, found on the tip facets, the relative number of exposed gold atoms on that surface is higher. This disparity encourages CTAB to attach more robustly to {100} facets, leaving {111} facets more accessible to incoming gold species, thereby fostering the anisotropic growth of the particle.^24^

Shortly thereafter, Mulvaney and colleagues proposed that variations in the electric field on the gold particle accounted for the anisotropic deposition of gold on the surface of both types of AuNRs. According to their theory, the Au(I) species, present as CTA^+^[AuCl_2_]^−^ complexes, combine with CTAB aggregates to form “metallomicelles”. The authors propose that the mobility of these structures determines the reduction rate of Au(I) species on the gold surface, with this process occurring more rapidly at the tips due to their higher electric field. This, in turn, triggers the rod shape during particle growth.^26^

Other studies have contributed to explaining the anisotropic growth of both single-crystal and pentatwinned AuNRs. They have shed light on crucial aspects of colloidal synthesis and offered detailed insights into the interaction between CTAB, the gold source, and the gold surface. Edgar and his colleagues demonstrated, using UV–visible absorption spectroscopy, that the gold precursor, HAuCl_4_, initially reacts with bromide ions. These ions are provided by the CTAB species, present in substantial quantities in the medium, thus forming [AuBr_4_]^−^ ions. These species are then reduced to [AuBr_2_]^−^ due to the presence of a mild reducing agent, ascorbic acid, in the growth solution.^27^

Hafner and his team used surface-enhanced Raman spectroscopy to validate the existence of Au(0)-Br^–^ interactions on the particle surface. They confirmed that the adsorbed CTAB could form either a compact bilayer or a “collapsed bilayer”, by monitoring changes in the number of interactions between the alkane chains of the CTA^+^ moiety and the AuNR surface.^28^

Because the crystallographic facets differ between the sides and tips of both single-crystal and pentatwinned AuNRs, the anisotropic growth mechanism was initially attributed to the selective adsorption of bromides on the highest energy facets.^29^ However, Vivek and Burgess performed electrochemical experiments on the adsorption of a quaternary ammonium bromide surfactant onto the {100} and {111} flat gold microplates. They demonstrated that there is no significant difference in the surfactants’ adsorption behavior onto the two crystal planes, despite varying levels of bromide adsorption. The authors concluded that there is no thermodynamic basis for supporting a model of preferential CTAB adsorption on different low-index gold facets.^30,31^ However, later, Wiley and his team conducted similar experiments, revealing that indeed, slightly different rates of gold deposition occur between Au{111} and Au{100} facets, with a ratio of approximately 1.46.^32^

## New Molecular Dynamics Insights into the Growth Mechanism of Gold Nanorods

**Figure 2:**
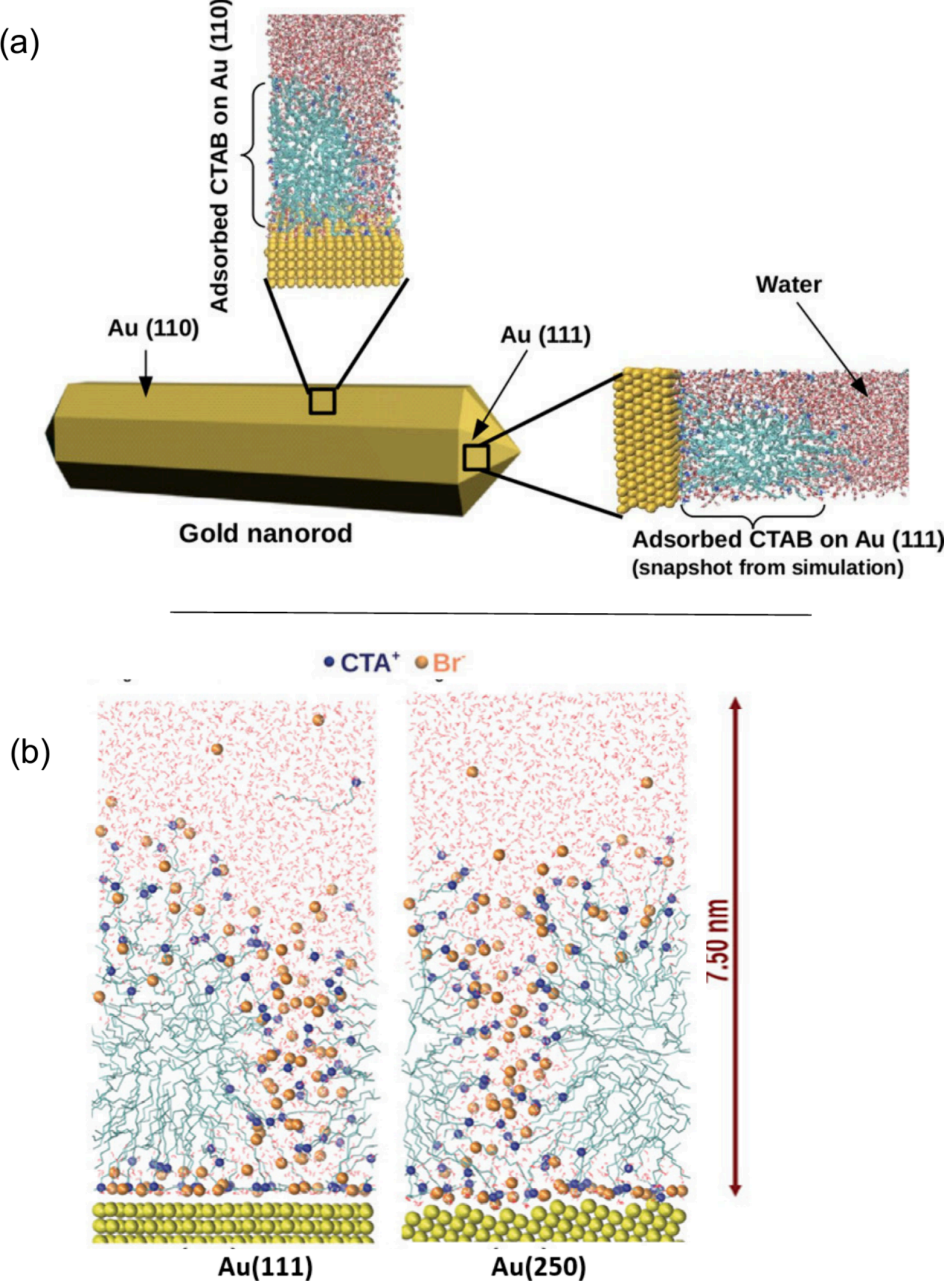
(a) Meena and Sulpizi simulation model. (b) CTAB adsorption on the gold surface with different crystallographic indexes. Copyright 2017 American Chemical Society.^33^ Copyright 2016 Wiley-VCH.^34^

Meena and Sulpizi^33−35^ pioneered the use of MD to investigate the anisotropic growth mechanism of AuNRs in colloidal synthesis. They established a simulation model representing the gold/surfactant/water interface (Figure 2a) to examine adsorption differences that could clarify anisotropic growth. Their findings suggest that quaternary ammonium bromide surfactant micelles attach to the surface of AuNRs, forming water–ion channels between adjacent micelles (Figure 2b), rather than the frequently described surfactant bilayer in the literature.^23,28^ The authors propose that these channels may facilitate the diffusion of Au(I) species toward the gold surface. They also noted that the thickness of these channels varies based on the facet where the micelles are anchored, with the largest channels discovered on Au{111} surfaces (0.94 nm) compared to Au{100} surfaces (0.73 nm). They further emphasized that the width of these channels is inversely proportional to the number of bromides adsorbed on the facets. Although this work does not delve into specific aspects related to the growth mechanism of single-crystal and pentatwinned AuNRs, it unveils innovative aspects of understanding the anisotropic growth of AuNRs based on MD simulations, paving the way for fresh experimental and simulation studies.

Another notable example of these developments is the theoretical work conducted by Sharma and co-workers. They utilized MD simulations to investigate the facet selectivity of CTAB on AuNPs.^36,37^ They demonstrated that the polar heads of surfactants, such as CTAB, interact preferentially with gold atoms situated between the facets of the nanoparticle, without any notable differences in interaction strength across different types of facets. In addition, they revealed that surfactants with longer carbon chains tend to interact more powerfully with each other than with the AuNP surface. These findings suggest that anisotropy arises due to the inhibition of lateral growth during the formation of AuNRs.^36^ They also explored different configurations of surfactants on metal surfaces and identified the most stable adsorbed structures for different surfactants. They discovered that Coulombic repulsion between ammonium head groups limits adsorption to a thin layer, whereas strong hydrophobic interactions among alkyl groups lead to the creation of adsorbed hemispherical micelles atop a monolayer.^37^ Interestingly, this hemispherical CTAB structure is consistent with thickness measurements of surfactant layers on AuNRs obtained through liquid microscopy.^38^

This lack of selectivity in CTAB’s interactions with the different types of flat gold surfaces was also observed in the simulations conducted by Meena et al.,^39,40^ even when polarization effects on gold surface^40^ were incorporated into their model.

Zeng and colleagues also used MD simulations to investigate the growth mechanism of silver nanoparticles in the presence of CTAB. They calculated the interaction energies between CTAB and the silver crystal planes {100}, {110}, and {111}, revealing that CTAB covers these surfaces with equivalent energies.^41^

This finding further underscores the concept that the anisotropic growth of nanoparticles is not primarily driven by selective surfactant interactions with specific crystallographic facets, but rather by other factors such as geometric and structural characteristics.

At this stage, it is worth noting that experimental data strongly suggest the presence of a compact CTAB bilayer on the surface of mature, single-crystal, or pentatwinned AuNRs.^23,28^ However, the proposed “*zipping* mechanism”^4,5^ assumes that this compact bilayer forms throughout the entire growth process. If a bilayer were present during this stage, it would imply that [AuBr_2_]^−^ and ascorbic acid species must leave the aqueous medium and spontaneously cross a hydrophobic barrier, a process that is likely unfavorable. However, as previously mentioned, one cannot disregard Hafner and co-workers’ proposed concept, based on experimental studies, that a “collapsed bilayer” of CTAB is observed during the early stages of AuNR formation.^28,42^

The mechanism proposed by Meena and Sulpizi is consistent with a process where both the lateral sides and tips of growing AuNRs develop, though at different rates. However, their initial simulation models were based on flat gold surfaces, thereby disregarding the curvature effect present on the sides and tips of AuNRs that could promote variations in intermicellar channel thickness. Additionally, a significant point to note in Meena and Sulpizi’s initial findings is that they did not observe the formation of a CTAB bilayer, a phenomenon frequently reported in the literature for mature AuNRs.

It is worth mentioning that, although the models for the lateral facets of AuNRs use a flat surface instead of a cylindrical, curved surface, we believe this simplification does not negatively impact the results. This belief is supported by the model’s ability to accurately reproduce the experimental properties of the adsorbed layer. Nonetheless, incorporating this refinement into the model could present an interesting avenue for future studies to explore.

Based on the foundational work of Meena and Sulpizi,^33−35^ Silva et al.^25,43,44^ conducted MD simulations to further explore the growth mechanism of AuNRs. For these simulations, we used the force field developed by Heinz et al.,^45^ proven to accurately represent the gold surface in contact with liquids and to reproduce adsorption energies. This model has also been successfully applied in simulations of nanoparticle growth for copper^46^ and silver^47^ in colloidal systems. In our simulations, electrostatic interactions were calculated using the generalized reaction field correction, applying a cutoff of 1.4 nm to both van der Waals and long-range electrostatic interactions. The simulations occurred under isothermal–isobaric (NPT) conditions, with periodic boundary conditions in all directions. The dimensions of our flat surface systems matched those used by Meena and Sulpizi: x = 4.0 nm, y = 4.0 nm, and z = 16.5 nm.^33,34^ The Berendsen thermostat kept the system’s temperature at 300 K, independently coupling the solute and solvent temperatures with a time constant of 0.4 ps.^48^ The Parrinello–Rahman barostat was used to control pressure,^49^ gently coupling the particle coordinates and box dimensions to a pressure bath of 1.0 bar.^50^ Pair-lists for short-range nonbonded and long-range electrostatic interactions were updated every five-time steps, and system configurations were recorded every 1 ps during the simulation. These lists identify which atoms are close enough to interact, serving as a foundation for calculating short-range interactions (e.g., van der Waals forces) and long-range electrostatic interactions. Updating the lists every five time steps, rather than at each step, is a strategic choice to reduce computational cost while preserving the accuracy of these interactions. The system’s state - specifically, position coordinates and velocities - was stored at 1 ps intervals for later analysis. This storing frequency strikes a balance, capturing enough data to monitor system trends over time without generating excessive amounts of data

In these simulations, we determined the packing density of CTA^+^ from the cross-sectional area of the CTA^+^ headgroup (0.32 nm^2^), in alignment with the experimental density of adsorbed CTAB layers.^51^ Each simulation lasted for 200 ns, offering comprehensive insights into the adsorption behavior and the structural organization of the CTAB layer on the gold surface.

**Figure 3:**
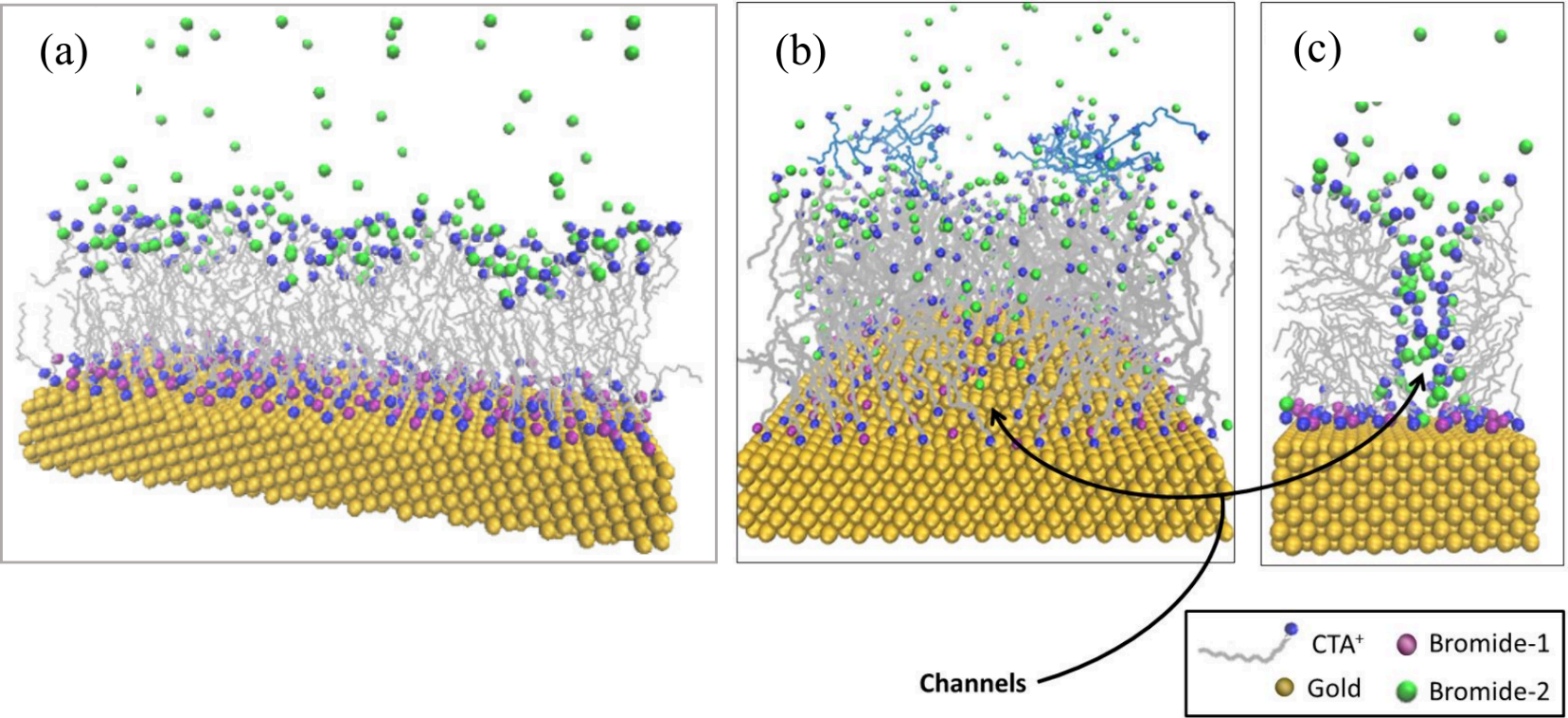
A surfactant structure on flat and curved surfaces. (a) Bilayer structure on a flat surface. Lateral view of the channels on (b) curved and (c) flat surfaces. Copyrights 2017 and 2020 American Chemical Society.^25,43^

Our results demonstrated that, under certain conditions, cylindrical micelles adjacent to or compact bilayer of CTAB could form around AuNRs - the outcome dependent on the relative concentration of bromide ions on the Au particle’s surface. A high concentration of bromide ions at the Au/CTA^+^ interface leads to the creation of a compact bilayer structure on the gold surface (Figure 3a).^25^ The bilayer, as seen in the simulation, has a thickness of 3.2 nm and presents interdigitation of apolar chains, which aligns with experimental measurements.^52^ We next evaluated the influence of curvature on the structure of the adsorbed layer under the same conditions as the flat surface,^43^ finding that it is more challenging to achieve a CTAB bilayer structure on a curved surface. This complication arises because some CTABs are not part of the adsorbed layer (see the blue chains in Figure 3b). In contrast, on a flat surface, all CTABs belongs to the adsorbed layer (Figure 3c).

This theoretical prediction implies that the CTAB density should be lower at the tips of AuNRs. This conclusion aligns with the findings of Huang et al.,^53^ who used Electron Energy Loss Spectroscopy in Aberration-corrected Scanning Transmission Electron Microscopy. It is also consistent with Bals and his team’s recent measurement of the CTAB layer thickness on single-crystalline AuNRs. They conducted the Electron Microscopy in a liquid environment using a graphene liquid cell.^38^ Therefore, comparing the simulated behavior on flat and curved surfaces reveals relative density differences between the two. This behavior can be extrapolated to the metallomicelle model, where the high mobility of the metallomicelle structure at the tips contributes to an increased reduction rate of Au(I) at these sites.

**Figure 4:**
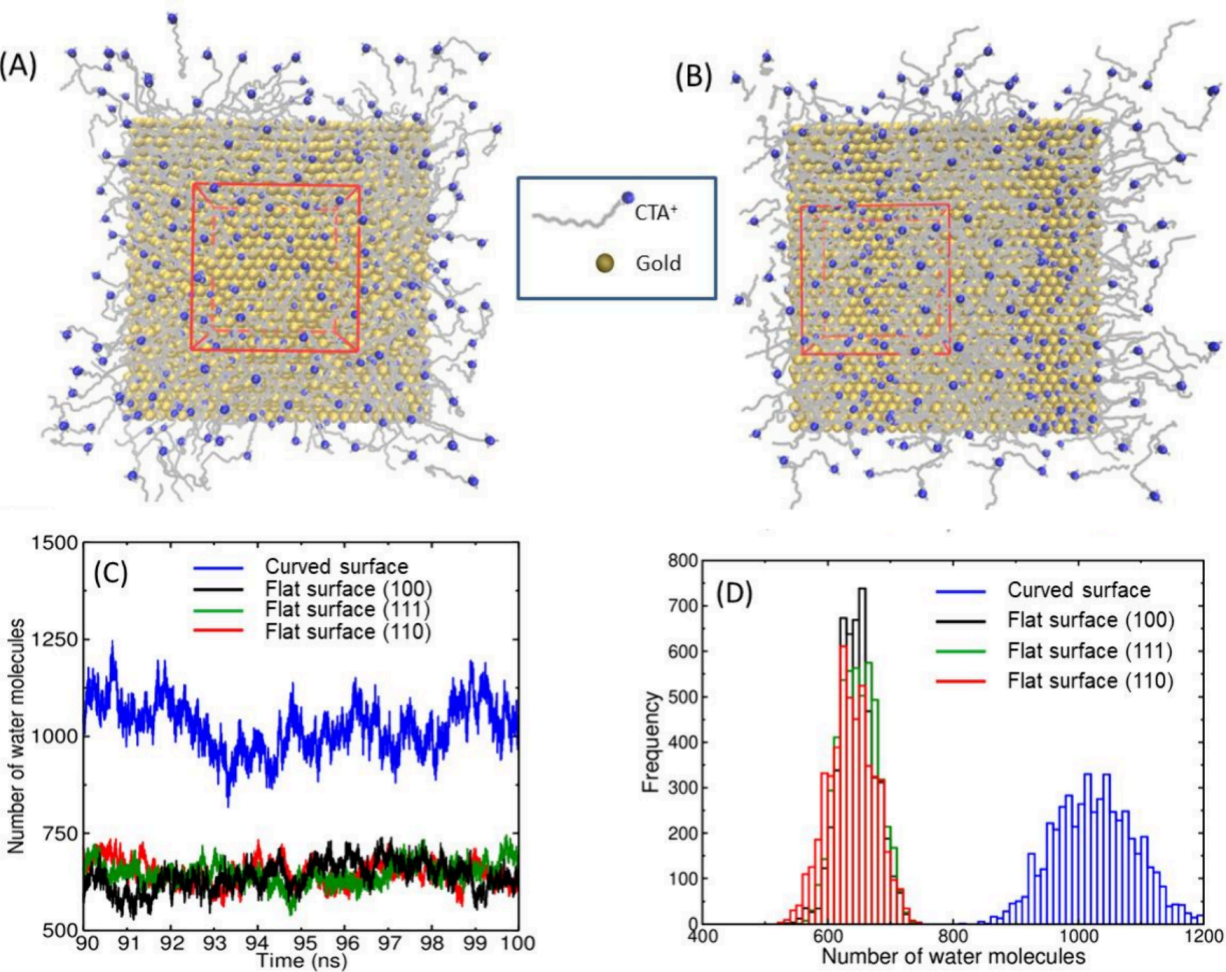
Snapshots of the top view (*z*-axis) of the MD simulations on a (A) curved surface and (B) flat surface (100). The water molecules and the bromide ions are omitted for clarity. (C) Number of water molecules inside the red box during the simulation. (D) Channel size measurement by the amount of water molecules inside the box. Copyright 2019 American Chemical Society.^43^

In our MD simulation studies, to assess the size and thickness of the intermicellar channels, we designated a cubic region (box) that encompasses a channel and tallied the number of water molecules inside the box when the micelles were anchored on flat or curved surfaces. Our findings suggested that the crystallographic index does not markedly influence the channel size or the design of the adsorbed layer on flat surfaces. However, curvature enlarges the channels (Figure 4).

**Figure 5:**
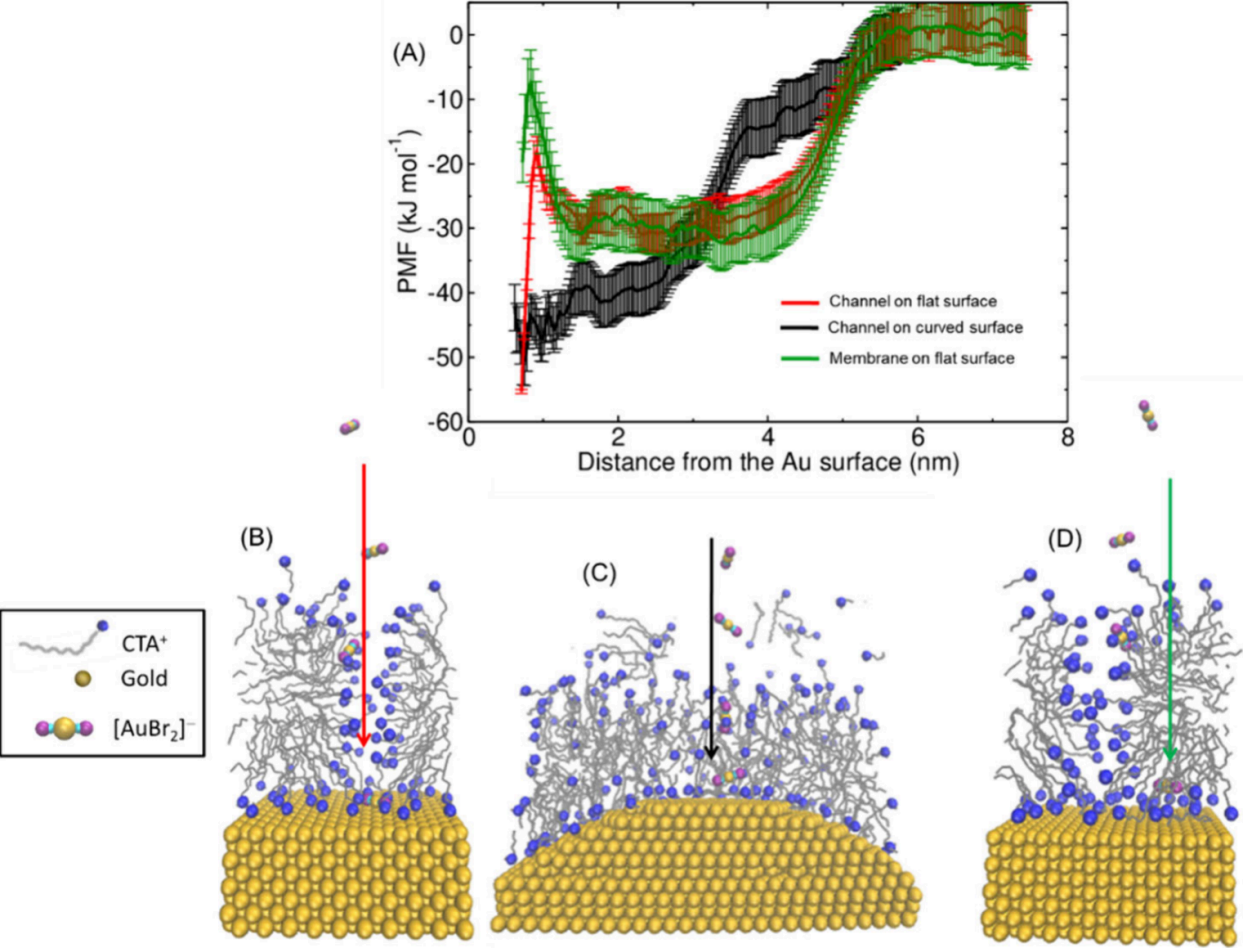
(A) Energy profile (potential of mean force, PMF) of umbrella sampling on different surfaces with a 5-site model. Passage of [AuBr_2_]^−^ through the channels on (B) flat and (C) curved surfaces. (D) Diffusion of [AuBr_2_]^−^ through the hydrophobic layer on a flat surface. The water molecules and the bromide ions are omitted for clarity. Copyright 2019 American Chemical Society.^43^

We conducted umbrella sampling simulations to measure the energy barrier for the passage of [AuBr_2_]^−^ species through channels present on both flat and curved surfaces, as well as across the membrane region (Figures 5A, 5B, 5C, and 5D).^43^ The [AuBr_2_]^−^ ion was pulled by the center of mass at a pulling rate of 0.001 nm ps^–1^, with a harmonic constant of 100 kJ mol^–1^ nm^–2^, from a starting position near the surface toward the bulk. Based on the snapshots generated from the pulling simulations, we executed and analyzed umbrella sampling simulations using the Weighted Histogram Analysis Method (WHAM).^54^ Our findings show that the energy barrier for Au(I) passage is lower on curved surfaces compared to flat ones, suggesting that curvature plays a significant role in the anisotropic deposition of gold species on the surface of growing AuNRs.

Based on these results, we propose a new growth mechanism for single-crystal AuNRs. In the initial stages of nanoparticle formation, CTAB micelles anchor to the metallic surface. Subsequently, following the symmetry break–attributed to the presence of silver ions in solution–larger channels form between the micelles on the tips of the developing AuNRs.^17^ This is a result of the increased curvature of the gold surface at these points. These expanded channels facilitate an easier passage of Au(I) species to the metallic surface, encountering lower energy barriers, thus elucidating the anisotropic growth of the nanoparticle. However, these channels disappear in mature AuNRs as compact CTAB bilayer forms, significantly limiting further nanoparticle growth in all directions.

**Figure 6:**
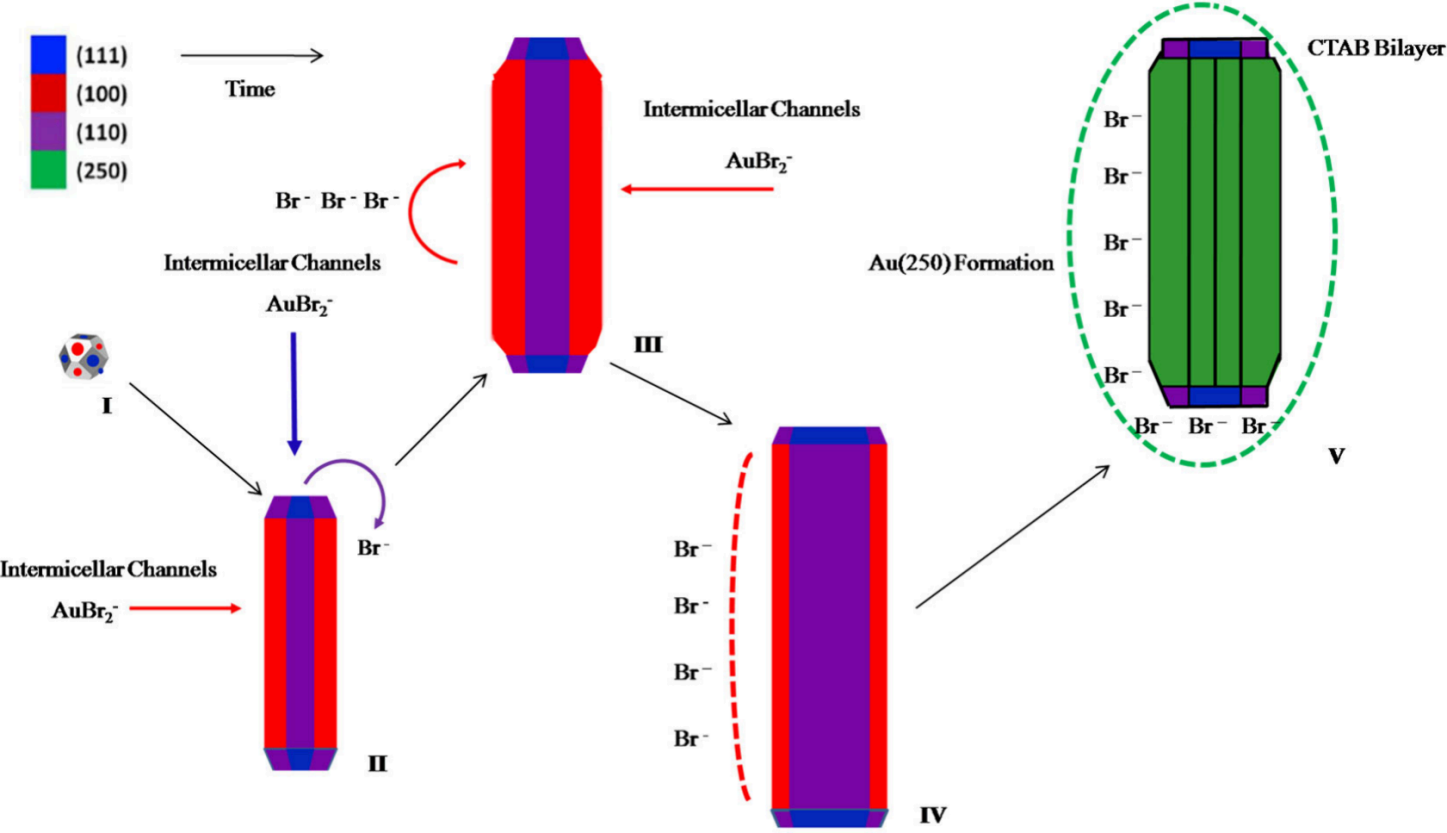
Illustration of the growth stages of a single-crystal AuNR during seed-mediated colloidal synthesis. Copyright 2017 American Chemical Society.^25^

The presence of a compact CTAB bilayer on mature AuNRs has been confirmed experimentally. We hypothesize that a transition from adsorbed micelles to a compact CTAB bilayer occurs during the growth process. It appears that this shift in CTAB arrangement on the particle’s surface is triggered by the accumulation of bromide ions at the Au/CTA^+^ interface as the AuNRs grow. This seems plausible since the reduction of [AuBr_2_]^−^ to Au(0) releases bromide ions locally (Figures 5 and 6).

## The Role of Silver Ions

The role of silver ions in the formation of single-crystalline AuNRs is still not completely understood. Typically, preparing these nanoparticles necessitates a significant amount of silver–about 10 mol % relative to gold–in the growth solution, although the final AuNRs only contain roughly 4 mol % of silver in their atomic composition.^55^ Nikoobakht and El-Sayed^5^ demonstrated that alterations in the concentration of silver ions in the growth solution significantly influence the AR of the resulting AuNRs. If the concentration of silver ions increases up to a certain point, an increase in the AR is observed. However, if these ions are absent from the reaction medium, no formation of AuNRs occurs.

Liu and Guyot-Sionnest^56^ have reported that in CTAB aqueous solution, Ag(I) ions can only be reduced to Ag(0) by ascorbic acid at pH levels above 8.0, whereas the typical pH of the growth solution in AuNR synthesis is around 5. Under these conditions, Ag(I) ions are not reduced to Ag(0), but can instead undergo underpotential deposition (UPD) onto the gold surface. Given the atomic arrangements in different gold low-index facets, the deposition of Ag(I) is expected to be more favorable on the {110} and {100} facets due to the higher coordination numbers (4) that Ag atoms can achieve on these facets, as opposed to the coordination number (3) attained on the {111} facet present on the tips. The authors suggest that the deposition of silver on these surfaces inhibits the reduction rate of Au(I) on the surface of the gold particle, thereby playing a role in the anisotropic growth of AuNRs.

Hubert and colleagues proposed that the CTA^+^[AgBr_2_]^−^ complex exists in the growth solution and adsorbs onto the gold surface, prompting the creation of rod-shaped particles.^57^ Almora-Barrios and associates used density functional theory (DFT) simulations to show that the CTA^+^[AgBr_2_]^−^ complex preferentially adsorbs onto the {100} facet as compared to the {111} facet, providing theoretical proof that this selective adsorption on the seeds results in the symmetry-breaking process.^58^ Later studies proposed that the complex present in the growth solution is CTABAg^+^, not CTA^+^[AgBr_2_]^−^, as shown by recent spectroscopic investigations, further confirming its importance in the synthesis mechanism.^59^ Xu and colleagues also identified the complex in the form of CTABAg^+^ and revealed that the presence of a minimal number of bromide ions in the growth solution of single-crystalline AuNRs is necessary to produce the complex and substantiated that a similar complex, CTACAg^+^, in the CTAC growth solution does not form. Based on this, the authors concluded that the CTABAg^+^ complex must play a critical role in the synthesis mechanism, though its exact role remains uncertain.^60^

A key point in the discussion of the AuNRs synthesis mechanism involves the process of seed symmetry breaking, which initiates the anisotropic growth of the particle. Walsh et al.^20,61^ demonstrated that in the synthesis of single-crystalline AuNRs, symmetry breaking is the result of the interaction between silver and the seed facets when the seeds are between 4–6 nm in diameter. It is noted that this symmetry-breaking process only takes place in seeds with a single-crystal structure; seeds containing twinned planes morph into gold nanospheres and other byproducts. Based on the observation by Tong et al.,^62^ the degree of symmetry breaking can be dictated by the [HAuCl_4_]:[AgNO_3_] ratio. This ratio then determines the AR of the resultant AuNRs. Interestingly, they also found that when silver is added after the symmetry-breaking event, there is a change in the width of the generated AuNRs, though the lengths astonishingly remain constant.

Moreau et al.^63^ explored the role of silver ions in the formation of single-crystalline Gold Nanorods (AuNRs). They demonstrated that during the early stages of AuNR growth, silver is preferentially deposited as Silver(0) onto high-energy surfaces like {110} and {100} facets, rather than {111}. The study also revealed that the silver distribution within AuNRs evolves over time, with the Silver/Gold coverage ratio decreasing as the growth process continues. It was shown that the growth rate on the sides of AuNRs is related to the presence of deposited Silver(0). Thus, the UPD hypothesis, proposed by Liu and Guyot-Sionnest^56^ was found to be consistent with the experimental observations.

## The Role of the Surfactant Counterion

**Figure 7:**
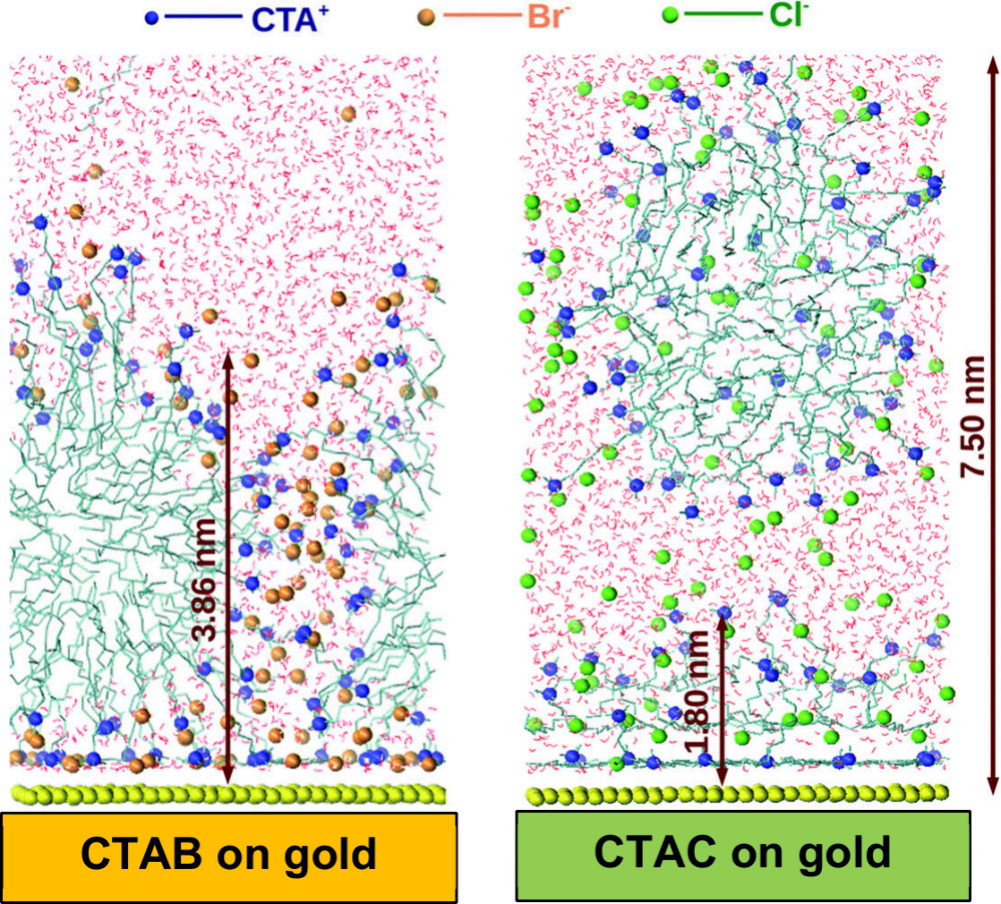
Counterion effect: different arrangements for CTAB and CTAC on the gold surface in aqueous solution. Copyright 2016 Royal Chemical Society.^35^

Several factors must be considered in the seed-mediated synthesis of AuNRs, with one of the most critical being the nature of the CTA^+^-counterion. Gold nanospheres tend to form instead of nanorods when chloride (CTAC) is used as the counterion as opposed to bromide (CTAB).^13,64^ MD simulations conducted by Meena and Sulpizi^35^ revealed that different counterions affect the thickness of the surfactant layer adsorbed onto the gold surface. Specifically, they confirmed that with CTAB, the surfactant layer remains intact on the gold surface, whereas with CTAC, this is not the case (Figure 7). Consequently, the authors argue that the synthesis of single-crystalline or pentatwinned AuNRs does not occur in CTAC because it cannot maintain a surfactant layer that protects and controls the rate of Au(I) reduction on the AuNP surface.

However, the literature indicates no substantial difference between the thickness of CTAB or CTAC layers.^65,66^ Indeed, the true difference between these two structures is related to the degree of counterion dissociation, which is about 0.24 (24% of ion dissociation) for CTAB and 0.38 (38% of ion dissociation) for CTAC.^67^ This discrepancy indicates that bromide ions maintain closer proximity to the polar headgroups of CTA^+^ than do chloride ions, and this decisively impacts how the micelles and the adsorbed layer behave. Lastly, the results noted in the simulation above contrast with the experimental findings of Sánchez-Iglesias and colleagues, who showed that the synthesis of pentatwinned AuNRs is possible with the growth solution having a combination of 98% CTAC and 2% CTAB.^68^ Xu and colleagues reached a similar conclusion, demonstrating that in the synthesis of single-crystalline AuNRs, the process can occur in CTAC as long as a slight amount of CTAB or a bromide source (0.15 mM) is present in the medium.^60^

Certainly, other factors can be examined to gain a deeper understanding of the mechanisms involved in the formation of AuNRs through seed-mediated methods. One such factor is the effect of modifying the cationic surfactant CTAB on AuNR development. As previously mentioned in the manuscript, the length of the larger alkyl chain in the surfactant,^12^ along with the size and chemical structure of the shorter alkyl substituents attached to the nitrogen in the polar head of the surfactant, significantly influences this process.^13−15^ MD simulations of these systems can offer useful insights into how surfactants interact with the gold surface and encourage anisotropic growth, ultimately determining the formation of AuNRs.

## Important Open Questions

Despite the considerable number and quality of research efforts dedicated to uncovering the mechanisms involved in the growth processes of AuNRs, many aspects remain unclear. While these gaps cannot yet be entirely addressed through simulations alone, simulation techniques can crucially contribute to advancing our understanding. An important approach involves refining the simulations, including the interaction of reducing agents at the interface, incorporating the CTABAg^+^ complex, and studying the mechanism from the perspective of metallomicelles. Other potential effects suitable for simulation include:

i) Investigating the role of the cosurfactant benzyldimethylhexadecylammonium chloride (BDAC) in the growth solution of the single-crystalline AuNR synthesis method. This approach significantly differs compared to the standard synthesis only using CTAB. In this situation, the cosurfactants presence results in AuNRs with a higher AR due to a reduced deposition rate on lateral facets and tips with significantly lower curvatures than those produced using the standard protocol.^8,69^
ii) Exploring the iodide effect in the synthesis of both single-crystalline and pentatwinned AuNRs. In the former case, trace amounts of iodide inhibit the formation of AuNRs,^70^ whereas in the latter, iodide increases the yields in terms of their formation;^71^
iii) Understanding why, under standard single-crystalline AuNRs production conditions,^72^ only 15% of Au(I) is reduced to Au(0).^55^ However, when more ascorbic acid is added in the reaction medium, complete reduction of Au(I) occurs, but the monodispersity of the resulting AuNPs depends on the way the addition occurs: a “dog-bone” is formed if the addition is fast and spherocylindrical geometry of the AuNRs is maintained if the addition is slow;^73^
iv) Wiley and colleagues further refined experiments initially performed by Vivek and Burgess.^30,31^ They also utilized gold microplates connected to electrodes in contact with solutions containing an Au(I) ion source and either CTAC or CTAB. Notably, they found no difference in the reduction and deposition of Au(I) on Au{100} or Au{111} facets when CTAC was used. However, in the presence of CTAB, they noted a 1.46-fold higher gold deposition rate on Au{111} compared to Au{100}.^32^ Despite being intriguing, this result sharply contrasts with the real-life synthesis of pentatwinned AuNRs, wherein the deposition rate difference is at least 10-fold higher. This stands in stark contrast with prior findings where the synthesis of pentatwinned AuNRs was achievable with a composition of 98% CTAC and 2% CTAB. Thus, this indicates the significance of additional factors, stretching beyond facet selectivity, during the anisotropic growth of pentatwinned AuNRs.

## Conclusions

In this review, our goal is to provide a general overview of the mechanistic aspects that underlie the growth and formation of AuNRs synthesized through seed-mediated methods. By integrating insights from experimental studies, quantum mechanics, and MD simulations, we aim to demonstrate how MD simulations can suggest, confirm, or reveal important aspects of the anisotropic nanoparticle growth process.

Despite the extensive research dedicated to understanding the synthesis of AuNRs through seed-mediated methods, many details remain unresolved. In both approaches—whether yielding single-crystalline or pentatwinned AuNRs, CTAB plays a role as a growth-directing agent. The interaction and arrangement of CTAB on the gold nanoparticle surface are key factors influencing the growth process. However, it is important to note that a single unified mechanism cannot be applied to both methods.

Notably, the anisotropy in single-crystalline AuNRs appears to result from the combined effects of tip surface curvature and the role of silver (via UPD and CTABAg^+^ complex). In contrast, the anisotropy of pentatwinned AuNRs does not seem to arise from selective adsorption on Au{100} versus Au{111} facets. Instead, it appears to be strongly influenced by the geometric characteristics of the tips, where twin planes are strategically aligned to facilitate the reduction of Au(I) species.

With respect to counterions, evidence suggests that bromide ions play a critical role in the symmetry-breaking process of the seeds, rather than significantly impacting the density or structure of the adsorbed surfactant layer. Additionally, the entire dynamic formation of the CTAB layer during AuNR growth appears more complex than the establishment of a simple double-layer arrangement around the lateral surfaces of the AuNRs. Instead, the process likely involves a micellar transition phase before the double-layer structure is achieved on mature AuNRs. Finally, this review aims to highlight the complexity of the mechanisms governing AuNR synthesis via seed-mediated methods, emphasizing the need for continued investigation to refine our understanding of these processes. Notably, even older simulations could be revisited and improved by applying updated calculation parameters.
